# Anti-COVID-19 Nanomaterials: Directions to Improve Prevention, Diagnosis, and Treatment

**DOI:** 10.3390/nano12050783

**Published:** 2022-02-25

**Authors:** Mohammad Souri, Mohsen Chiani, Ali Farhangi, Mohammad Reza Mehrabi, Dariush Nourouzian, Kaamran Raahemifar, M. Soltani

**Affiliations:** 1 Department of NanoBiotechnology, Pasteur Institute of Iran, Tehran 13169-43551, Iran; souri1996m@gmail.com (M.S.); chiani@pasteur.ac.ir (M.C.); farhangi@pasteur.ac.ir (A.F.); 2 Department of Mechanical Engineering, K. N. Toosi University of Technology, Tehran 19967-15433, Iran; 3 Data Science and Artificial Intelligence Program, College of Information Sciences and Technology (IST), Penn State University, State College, PA 16801, USA; kraahemi@gmail.com; 4 Department of Chemical Engineering, University of Waterloo, 200 University Avenue West, Waterloo, ON N2L 3G1, Canada; 5 School of Optometry and Vision Science, Faculty of Science, University of Waterloo, 200 University Avenue West, Waterloo, ON N2L 3G1, Canada; 6 Department of Electrical and Computer Engineering, University of Waterloo, Waterloo, ON N2L 3G1, Canada; 7 Centre for Biotechnology and Bioengineering (CBB), University of Waterloo, Waterloo, ON N2L 3G1, Canada; 8 Advanced Bioengineering Initiative Center, Multidisciplinary International Complex, K. N. Toosi University of Technology, Tehran 14176-14411, Iran

**Keywords:** SARS-CoV-2, nanotechnology, prevention, diagnosis, treatment

## Abstract

Following the announcement of the outbreak of COVID-19 by the World Health Organization, unprecedented efforts were made by researchers around the world to combat the disease. So far, various methods have been developed to combat this “virus” nano enemy, in close collaboration with the clinical and scientific communities. Nanotechnology based on modifiable engineering materials and useful physicochemical properties has demonstrated several methods in the fight against SARS-CoV-2. Here, based on what has been clarified so far from the life cycle of SARS-CoV-2, through an interdisciplinary perspective based on computational science, engineering, pharmacology, medicine, biology, and virology, the role of nano-tools in the trio of prevention, diagnosis, and treatment is highlighted. The special properties of different nanomaterials have led to their widespread use in the development of personal protective equipment, anti-viral nano-coats, and disinfectants in the fight against SARS-CoV-2 out-body. The development of nano-based vaccines acts as a strong shield in-body. In addition, fast detection with high efficiency of SARS-CoV-2 by nanomaterial-based point-of-care devices is another nanotechnology capability. Finally, nanotechnology can play an effective role as an agents carrier, such as agents for blocking angiotensin-converting enzyme 2 (ACE2) receptors, gene editing agents, and therapeutic agents. As a general conclusion, it can be said that nanoparticles can be widely used in disinfection applications outside in vivo. However, in in vivo applications, although it has provided promising results, it still needs to be evaluated for possible unintended immunotoxicity. Reviews like these can be important documents for future unwanted pandemics.

## 1. Introduction

**Figure 1:**
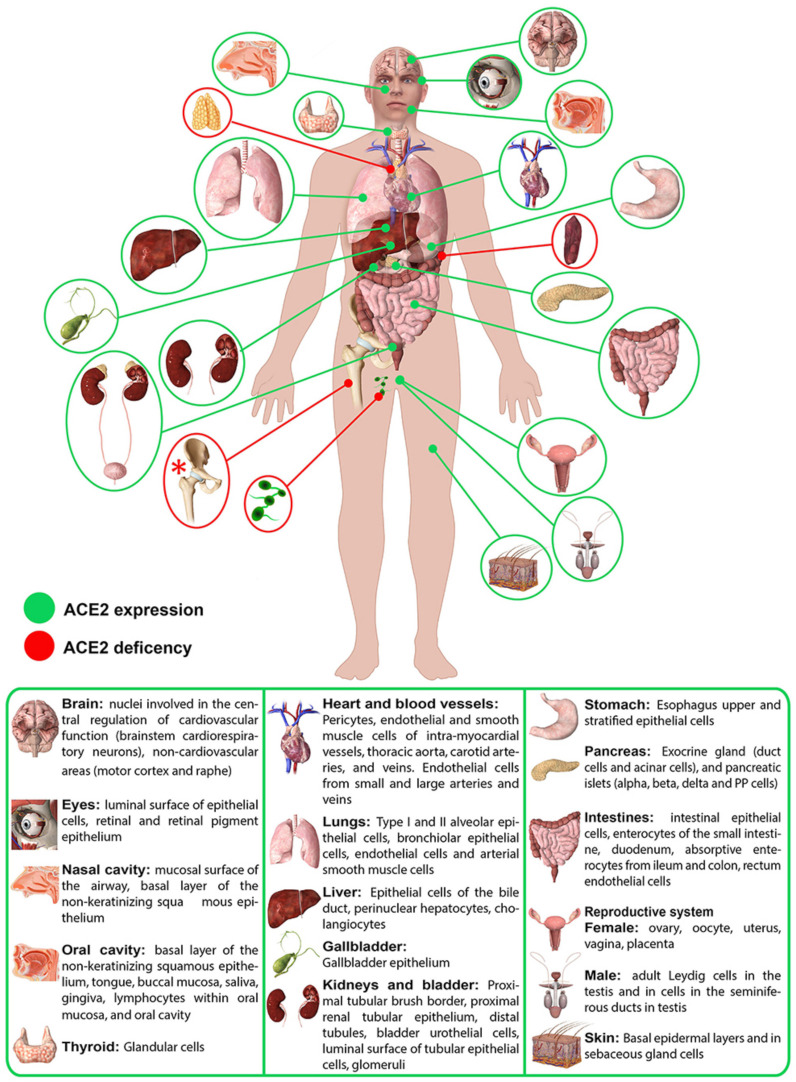
Schematic of the distribution of ACE2 in human organs; it is clear that the major organs that provide ACE2 are therefore all susceptible to SARS-CoV-2 infection. According to the WHO, many patients have been referred to hospitals complaining of pain in various organs following involvement with COVID-19. Reproduced with permission from [19]. Copyright Frontiers, 2020.

In March 2020, the World Health Organization (WHO) announced a pandemic disease called COVID-19, which is caused due to severe acute respiratory syndrome coronavirus 2 (SARS-CoV-2) [1]. The disease is now affecting people all over the world, from crowded cities to the most remote tribes in the deserts and forests. In the current century, the human coronavirus family has caused four other outbreaks, including SARS [2], MERS [2], Ebola [3], and swine flu [4]. Deaths from these four outbreaks have been less than 25,000 [5], so they were not enough alarms for a pandemic like the current coronavirus pandemic. COVID-19 is a bully threatening life and public health, which has become a heavy economic burden around the world. Many governments and politicians have continued to resort to social distancing and home quarantine, as well as the therapeutic methods against COVID-19, given the new variants of COVID-19, which have a much higher incidence rate. Factors such as the patient’s age, the patient’s clinical condition, the availability of intensive care, and the location prevent the accurate determination of case mortality for COVID-19 [6]. However, according to the WHO, at the time of submission of this review paper, more than 364.19 million people have been diagnosed with COVID-19 and more than 5.63 million have died [1]. Given the advancement of technology and biology in the current decade, this is a disaster. The size of this virus is 65 to 125 nm, which according to the evidence, the most important way of transmission is through penetration of infected droplets (of diameter > 5 μm) to mucous surfaces with and contact with the infected surface [7,8]. The likelihood of airborne transmission is very low unless droplets less than 5 μm in diameter are contaminated with the virus and remain in the air for longer periods [9]. The disease varies from person to person, from mild respiratory illness to acute respiratory syndrome. COVID-19 infections can also affect various organs in the body, including the central nervous system [10,11], cardiovascular system [12], kidneys [13], gastrointestinal tract [14], and liver [15,16]. Furthermore, a common cause of death is an uncontrolled cytokine, which can lead to stroke due to blood clots, organ failure, and heart attacks [17]. Due to SARS-CoV-2 must bind to angiotensin-converting enzyme 2 (ACE2) to enter host cells in humans, the expression and body localization of ACE2 is important [18]. It has recently been reported that ACE2-expressing organs can become direct targets of SARS-CoV-2, leading to severe pathobiological manifestations followed by multiple organ failure (Figure 1) [19].

There are currently three major challenges to COVID-19, including prevention, early diagnosis, and treatment. So far, various vaccines have been introduced for prevention, some of which have been very promising in the clinical phases, but only one has been approved by the FDA (the Pfizer–BioNTech COVID-19 vaccine [20]) [21,22]. However, there are still concerns that if new variants emerge, will the vaccines introduced be effective against them? Available treatments are mostly used to reduce the severity of the disease and save lives which are based on non-specific antiviral drugs and symptomatic treatment, such as remdesivir [23], Ivermectin [24], and hydroxychloroquine [25]. However, hydroxychloroquine has failed in the clinical phase due to severe side effects as a potential treatment for SARS-CoV-2 [26]. The other two cases need further evaluation. Therefore, basic preventive measures such as using a special mask, social (physical) distance, and practicing hygiene are still necessary. Determining the prevalence in the early stages is also a concern that great efforts have been made in the field of early diagnostic measures such as rapid and advanced tests, tests based on artificial intelligence, and computed tomography (CT) scans [8]. Standard methods of treatment and vaccination are also mainly by targeting key processes in the virus life cycle. However, many viruses, especially current viruses, can evolve and become resistant to drugs under selective pressures. Hence the development of resources is essential for the production of new therapeutic agents.

**Figure 2:**
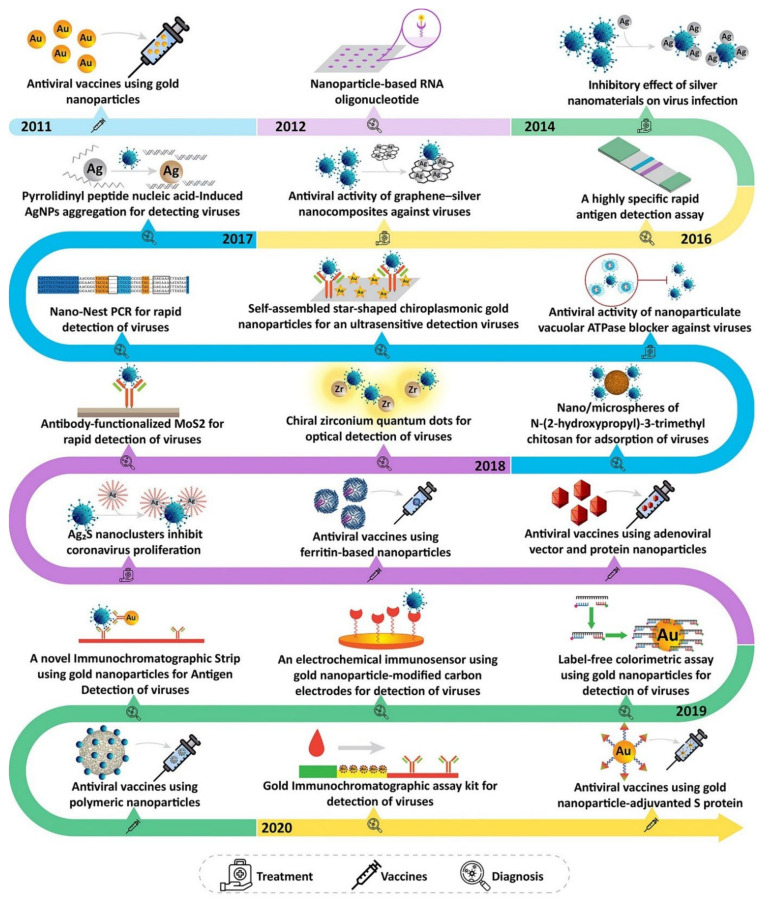
The effective role of nanoparticles in inhibiting viruses as nano-vaccine components, biosensing, and nanomedicine. Reproduced with permission from [33]. Copyright American Chemical Society, 2021.

Multidisciplinary features COVID-19 provided a platform for nanomaterial researchers to take action and play an important role. Nanotechnology, as an interdisciplinary field focused on translation, is able to solve many problems by engineering solutions and reducing the pressure already placed on medical centers [27]. The advantage of nanotechnology is that it operates on the scale at which viruses such as COVID-19 operate. At this scale, structures are about 10 times larger than individual atoms, so easy interaction with microorganisms accelerates the development of diagnostic and therapeutic methods in the biomedical field [27]. The effective role of nanoparticles in inhibiting viruses has already been proven (Figure 2). Nanoparticles combined with increased bioavailability can effectively deliver drugs and genes to target cells and tissues without being exposed to external hazards [28]. Recently, engineered nanoparticles coated with specific proteins, have introduced new treatment options, an approach that is not available for other therapeutic purposes [29,30]. Engineered nanoparticles can focus on damaged cells, thereby minimizing cell infection [29]. Nanoparticles are also able to deliver antiviral agents at any stage with controlled release, while boost immune system vaccines intervene only at a certain stage of the virus replication cycle [27,31]. Nanomedicine can be very promising against COVID-19. The multivariate nature of nanomedicine such as drug delivery, diagnosis, and theranostics can provide a catalyst for new technologies in therapy [32].

**Figure 3:**
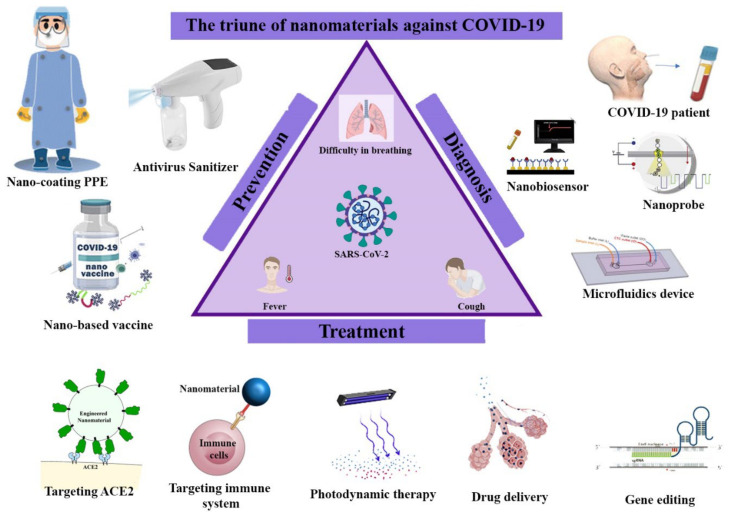
An overview of what is being reviewed in this work; First, the virus prevention approach is reviewed, which includes surface disinfectant sprays, antiviral coatings, and the development of vaccines based on nanotechnology. The Section 2 reviews nanotechnology-based platforms for virus detection. The Section 3 also deals with treatment based on nanotechnology which includes various methods including drug delivery, gene editing.

Nanomedicine has already proven its value in other diseases such as cancer. Given the millions of infected people and the rising mortality rate, as well as the rapid global transmission of the Coronavirus, we are looking to explore the use of nanotechnology in preventive diagnostic and therapeutic applications. One of the important advantages of this review is that it is written, from the perspective of people with interdisciplinary specialties, active in the interdisciplinary field of nanotechnology, against an interdisciplinary pandemic. The authors try to discuss the gaps and possible opportunities for managing and treating COVID-19 based on the science of nanotechnology. In particular, we highlight the use of different nanomaterials such as metal nanomaterials and organic nanomaterials in various anti-COVID-19 applications. This review can be a comprehensive document on the applications of nanotechnology versus COVID-19 for future studies. An overview of what is being reviewed in this work is provided in Figure 3.

## 2. Prevention

Deaths from COVID-19 continue to rise alarmingly in some countries. Prevention strategies from the current pandemic include vaccines, antiviral drugs, and non-pharmacological countermeasures. Non-pharmacological countermeasures should be considered as an important approach as many countries do not have adequate sources of medicines and vaccines to prevent SARS-CoV-2 infection. Since the most important way of transmission is through aerosol droplets, it is necessary to use a facemask in public places, take care of contaminated hands and follow the usual hygienic methods [34]. Pathogens such as SARS-CoV-2 can remain on inanimate surfaces for more than 9 days [35]. Therefore, disinfectants are used to remove pathogens from surfaces. Given the various methods of transmitting COVID-19 that are known so far, and there may be other unknown ways, prevention is the most important way to deal with COVID-19, which can include disinfection of surfaces and the environment, personal protection equipment (PPE), and vaccinations [36,37,38,39,40,41,42,43,44,45]. There are currently a variety of preventative tools on the market. In this regard, nanotechnology offers new opportunities for the development of COVID-19 prevention approaches that offer high sterilization with low doses, no side effects, user-friendliness, and longer life is its most important features [46,47,48]. The following is the use of nanomaterials as surface disinfectants, applications in PPE, and delivery systems for the development of prophylaxis vaccines.

### 2.1. Sterilization and Disinfection of Inanimate Surfaces

Unlike other viruses, SARS-CoV-2 has a higher resistance so that it can remain active on inanimate surfaces such as fabric, metal surfaces, wood, plastic, glass, and skin for several hours to several days [49,50]. This leads to its high prevalence in public places. Hence daily disinfection of surfaces is necessary. Disinfectants on the market are mainly based on alcohol and its derivatives. These disinfectants can eventually protect the user or surfaces for up to a few minutes [51,52]. Therefore, the surfaces can be a place for the virus to accumulate again. The corrosive nature of alcohol, low user-friendliness, and poor environmental compatibility have made nanotechnology seize the opportunity and provide a platform for the development of disinfectants. Nanoparticles with less than 20 nm have been reported to be more dependent on pathogens, causing pathogen death [53,54,55]. In the following, the application of nanoparticles in disinfectants is investigated (Table 1).

#### 2.1.1. Metal Nanoparticle-Based Disinfectant

Due to their antiviral and antibacterial properties, metal nanoparticles have a variety of applications, including disinfectants [63,64,65]. Metal particles such as silver and copper, unlike alcohol-based disinfectants, are environmentally friendly, non-flammable, and non-volatile referred to as green technologies [66,67]. Metal nanoparticles act mainly on the surface of the virus and prevent the physical interaction of viruses and host cells. This is very valuable because the infection of the host cell by the virus occurs with the entry of the nucleic acid of the virus into the host cell after physical contact [8]. Metal nanoparticle-based disinfectants have advanced safety and health properties that can be promising against COVID-19. Various companies have developed disinfectants based on metal nanoparticles. SHEPROS SDN BHD has developed a nanosilver-based multipurpose disinfectant using a nano-colloidal technique that has environmentally friendly, non-irritating, and non-foaming properties to fight viruses, germs, and fungi [56]. An Italian-made nano-sterilizing product by NanoTechSurface, a solution containing silver ions and titanium dioxide has been developed to disinfect surfaces contaminated with SARS-CoV-2 [57]. Concerns about metal nanoparticles are the toxic effects of metal nanoparticles that can be reduced by the use of biodegradable nanomaterials such as polymers and lipids [8,57]. Based on this, an independent startup in Pune (Weinnovate Biosolutions) has developed disinfectants based on silver nanoparticle solution [58].

#### 2.1.2. Naturally Nanomaterials-Based Disinfectant 

Natural nanoparticles are very popular due to their high environmental friendliness, are non-corrosive, non-toxic, cost-effective, and user-friendly, and are made using various techniques from natural sources such as insects, animals, flower leaves, and fruits [68]. These substances mainly act as adsorbents in sanitizers, which release disinfectants molecules in a controlled release. In this type of disinfectant, due to the greater interaction of disinfectant molecules with existing viruses, the percentage of protective and disinfecting capacity increases [8,52,69]. Recently, Tamil Nadu University has introduced a natural nanomaterial-based disinfectant that is able to release hydrogen peroxide and alcohol molecules in a steady and sustained release within 20 to 25 min [8]. There is also a disinfectant called Ananya reported by the Defence Institute of Advanced Technologies (DIAT) [59]. This disinfection is based on water spray using nanotechnology, which is able to adhere to fabric, plastics, and metal surfaces, so Its disinfection effects may about 6 months.

#### 2.1.3. Nanopolymer-Based Disinfectants

Nanopolymer-based disinfectants have had antimicrobial properties for a long time, are user-friendly, environmentally friendly [8,70]. Design.123 has developed a product (PRELYNX PORTAL) used to disinfect environments contaminated with COVID-19, which works on nanopolymer-based applications and inactivates hydrophilic and lipophilic viruses in 20 min of contact [60]. Recently, a temperature-responsive polymer-based antimicrobial coating has been developed by the Hong Kong University of Science and Technology (HKUST), which by slowly releasing disinfectants is able to inactivate the lipid envelope of viruses and prevent virus adhesion for up to 90 days [61].

#### 2.1.4. Light-Activated Nanocoating Disinfecting Viruses

Light-activated nanomaterials are mainly composed of inorganic nanocrystals that kill microbes at appropriate wavelengths [71,72]. The interaction of nanocrystals causes a strong oxidation reaction that decomposes any pathogen on the surface. The antimicrobial mechanism of this strategy includes photocatalytic disinfection, photothermal, and photodynamic killing, which damages and destroys the protective membranes and genetic material of the virus [73,74,75,76]. NanocleanSQ is an antiviral coating created by Canadian scientists. This coating is able to kill all viruses in case of contact and its lifespan is up to a year [62]. NanoTouch Materials, LLC, has also reported a nano-coating based on mineral nanomaterials that destroy the virus by an oxidation reaction after getting charged by visible light [8]. The photodynamic activity of nano-coatings (based on TiO_2_) also reduces the spread of infections caused by COVID-19 [77]. In general, modifying intact surfaces such as fences, bed surfaces, and handles with nanomaterials can keep germs and viruses away.

### 2.2. Nano-Based Disinfectant Personal Protective Equipment (PPE)

**Figure 4:**
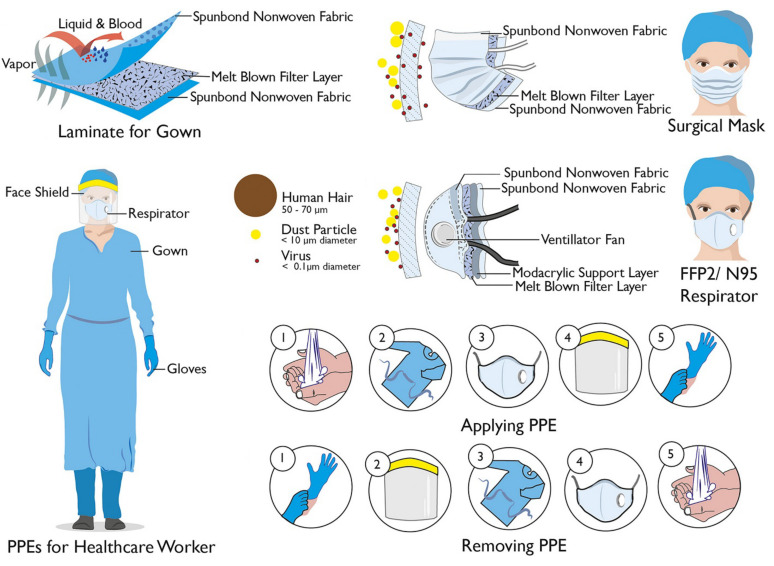
PPE for health care workers; safe PPEs for health care workers include visor respirator, visor, gloves, and gown. FFP2/N95 respirator and surgical mask provide protection against airborne viruses and larger particles, respectively. A disposable medical gown provides protection from blood and liquid. At the same time, the steps of placing and removing PPEs are very important for regulating health care. Reproduced with permission from [84]. Copyright American Chemical Society, 2020.

In general, if the users are in less contact with the COVID-19 virus, they are less likely to be infected. The most important protection measures used by ordinary people and front-line health care workers are PPE (Figure 4). One of the most basic methods of prevention against COVID-19 is to cover the face with a mask [78,79]. Mask is essential for non-infected and infected people, infected people can prevent the spread of the virus by covering their face, and uninfected people can protect themselves against the COVID-19 virus by covering their faces [80,81]. The most commonly used face masks are called N95, which can filter 95% of contaminants in a certain size [82,83]. However, the COVID-19 virus is capable to retain on the surface of the textile. In general, PPE is not an anti-virus or anti-bacterial and needs to be replaced constantly. There have been recent reports of nanotechnology-based innovations that the use of washable anti-viral nano-coatings can enhance the protective capabilities of COVID-19 (Table 2).

#### 2.2.1. Nanoparticles-Based PPE

Various metal nanoparticles, such as copper, gold, silver, have great potential for making masks and personal PPE due to their antimicrobial and antiviral properties [69,92]. Recently, nanosilver coatings have been used to make natural three-layer masks [93]. It has also been reported that a spray based on copper and silver nanoparticles has been installed on the face mask and PPE to increase protection [94]. Promethean Particles Ltd. partnered with a textile company to create a fabric based on copper nanoparticles embedded in a polymer matrix [85]. These fabrics are for enhancing antiviral and antimicrobial properties for the healthcare sectors. Sonovia Ltd. has also developed a fabric based on zinc oxide nanoparticles that in addition to its antiviral properties, can be washed for reuse. This fabric is used for masks (called Sonomasks) and PPE [86,87]. A coating based on ultraviolet light-responsive nanomaterials has been developed by Park et al., Which performs a chemical reaction in the presence of a light stimulus to kill viruses [8,86]. Another attractive coating based on nanodiamonds that can be used in masks and PPE as well as ventilators has been introduced by Master Dynamic Limited [86]. This coating has a high anti-virus performance that can even destroy it. X.TiO2 Inc. (XTI) claims that TiO_2_Ag-based facemasks are able to kill 99.99% of viruses under zero light conditions [86]. This mask is refreshed in exposure to direct sunlight and kills all accumulated viruses on the surface of the mask. What was found was the strength of nanoparticles, especially metal nanoparticles, in inhibiting the coronavirus. However, what is worrying is the inhalation of metal nanoparticles, especially copper oxide, which may cause adverse toxic reactions [8,17,26,79]. Therefore, metal nanoparticles in integration with PPE should be highly considered and analyzed.

#### 2.2.2. Nanofibrous Membrane-Based PPE

Nanofiber membrane which forms based on a dense weblike network of nanofibers is a very high effective surface area against infect. These membranes are mainly included in facemasks that provide high breathing and filtration efficiency. Nanofibers act in such a way that nucleating agents and chemicals, p-iodobenzoic acid and ß-cyclodextrin, activate nanofibers against pathogens and reduce their risk of inhaling by degrading and inactivating pathogens and viruses [46,95]. The synthesis of nanofiber-based materials is based on the standard electrospinning technique, which increases the absorption of target particles by generating an electric charge [96,97]. Ultrasonic technology is also used to assemble the facemask. Ultrasonic technology allows bonds to be created quickly to seal seams and edges for mask production [98]. Recently, nanocellulose nanofibres product from waste plant material has been developed by T. Rainey et al., Which claim to protect from pathogens of size up to 100 nm [8]. Kim et al., created nanofiber-based filter masks that are water-resistant and non-deformable, with a 94% filtration efficiency [99]. YAMASHINFILTER CORP claims that nanofiber-based nanoresin masks have thermal and antiviral properties [8]. The Amrita Centre for Nanosciences and Molecular Medicine (ACNSMM) has developed a cost-effective disposable mask that possesses two layers of biodegradable fabric based on nanococo-carbon fibers [8,100]. The center claims that this mask is able to prevent 99.99% of pathogens from entering the body. Nanopoli, Korea, has developed a nanofiber-based mask that has a variety of layers, including a water repellent and a skin-friendly silk layer with a 98% filtration guide [8].

### 2.3. Vaccination

The best way to fight the virus is to prevent it from entering the target body. However, many people are unwittingly exposed to the virus, in which case the clearance must be done before the virus can infect the target cells or spread throughout the body. Vaccines allow for such a type of intervention because they can prevent infections caused by viruses such as COVID-19. However, vaccines can take months or even years to develop and become available to the public. The mechanism of action of the vaccines is that after injection, it gives the immune system a preview of the virus without causing the disease, which causes the immune system to be informed of the virus characteristics. In the event of a real virus, the immune system detects and fights a virus that contains known characteristics [27]. The immune system mainly detects viruses with virus unique proteins. Vaccines are currently based on killed viruses, whole-cell live attenuated vaccines, gene-based vaccines, and subunit vaccines [101]. Challenges that must be considered in the development of vaccines include efficacy, immunogenicity, vaccine safety, and risk of infection [17]. Previous knowledge of immune responses and the similarity of SARS-CoV-2 to other viruses, including SARS-CoV and MERS-CoV, has greatly contributed to the development of COVID-19 vaccines.

**Figure 5:**
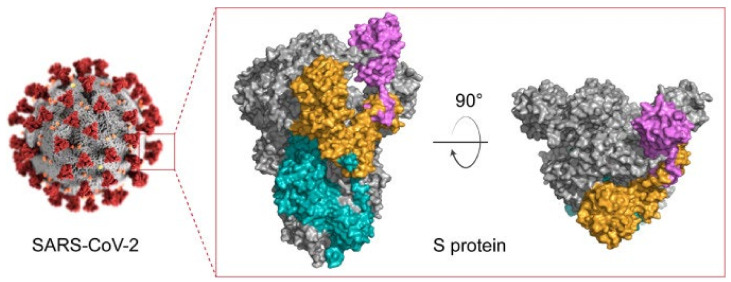
SARS-CoV-2 S protein consists of three identical chains with different colors of yellow that demonstrate S1 domains, and cyan that demonstrate S2 domains. Reproduced with permission from [108]. Copyright Elsevier, 2021.

COVID-19 virus is an enveloped ssRNA virus, which has spike-like glycoproteins that protrude from the surface of the virus membrane forming a ‘corona’. Spike (S) protein, Nucleocapsid (N) protein, Membrane (M) protein, and Envelope (E) protein are the structural proteins of beta coronaviruses [102]. The design of vaccines is mainly based on the S protein because this protein facilitates the entry of the virus into the host cell to cause infection. The spike protein has two important subdomains; (i) the S1 subdomain, which includes the receptor-binding domain (RBD) that is responsible for binding the angiotensin-converting enzyme 2 (ACE2) to the host cell, and (ii) the S2, which is called the fusion machine and is responsible for fusion with the host cell membrane which facilitates the entry into the cell (Figure 5) [103]. In different types of coronaviruses, the S1 domain is divergent, while the S2 domain is more conserved [104]. Combining and comparing recorded data from the SARS-CoV-2, knowledge of SARS/MERS vaccines, S proteins information, and its S1 and S2 subdomains, have helped researchers to obtain neutralizing antibodies. Neutralizing antibodies mainly target different domains of the S protein. Recent clinical data from a cohort have shown that analysis of patients’ sera indicates that S1 and S2 are targeted by neutralizing antibodies [105]. Based on computational analysis, glycosylated SARS-CoV-2 S protein has a more organized structure than its non-glycosylated counterpart [106,107]. Therefore, glycosylation of SARS-CoV-2 vaccine should be considered.

Intense efforts and research have been carried out to develop vaccines against COVID-19, so this extraordinary scientific mobilization introduced the first vaccine candidate to enter human clinical trials on, 16 March 2020 [109]. In addition to the FDA-approved Pfizer-BioNTech vaccine, about 10 other vaccines have been successful in their clinical evaluations, and about 114 more are in the preclinical development phase [110]. In the development and research of vaccination, nanotechnology can provide a platform to act on antigens specifically by directing the immune response.

#### 2.3.1. Nano-Based Approach for COVID-19 Vaccine

Nanomedicine can be a key component in the synthesis of vaccines to boost the immune system or it can be involved as a carrier of the active agent in vaccines against COVID-19 [111,112]. Important properties of nanoparticles for antigen delivery, such as changes in physicochemical properties, overcome problems such as limited immunogenicity and reversion to pathogenic virulence, which is very common in conventional and subunit vaccines [5]. In general, the purpose of selecting nanoparticles in vaccine formulations can meet three important issues: (i) targeted delivery of antigens, (ii) enhanced immunogenicity, and stimulation of the immune response, and (iii) protection of antigens against premature destruction by proteolytic enzymes [113].

Biocompatible nanoparticles such as polymers, liposomes, lipid nanoparticles, and emulsions are non-toxic nanoparticles that can increase the solubility and stability of vaccines. Modification of the nanoparticle surface with toll-like ligands such as mannose and immune cell target ligands increases vaccine efficacy by facilitating targeted delivery of vaccine cargo [114]. Among the COVID-19 vaccines produced based on nano, ‘Pfizer’ and ‘Moderna’, used mRNA nanoparticles with lipid nanocarriers. Nanocarriers protect their cargo against biodegradation by the immune system and deliver it inside the cell membrane. Once the cargo is released, the bare mRNA encodes the protein antigen and provides immunity against SARS-CoV-2 [114]. The second strategy, which is based on immunity by encoding the SARS-CoV-2 spike protein, has been used by ‘Oxford-AstraZeneca’ and ‘Sputnik V’ to develop a vaccine against COVID-19. This strategy is based on non-replicating adenovirus [115]. Multifunctional approaches of nanomaterial can enhance the bioavailability, controlled antigen release, and specific immune activities [115]. In general, the use of nanomedicine in the development of the COVID-19 vaccine is based on two strategies: antigen delivery and adjuvant to improve the immune response [112]. Table 3 summarizes the role of nanotechnology in vaccine developments.

**Figure 6:**
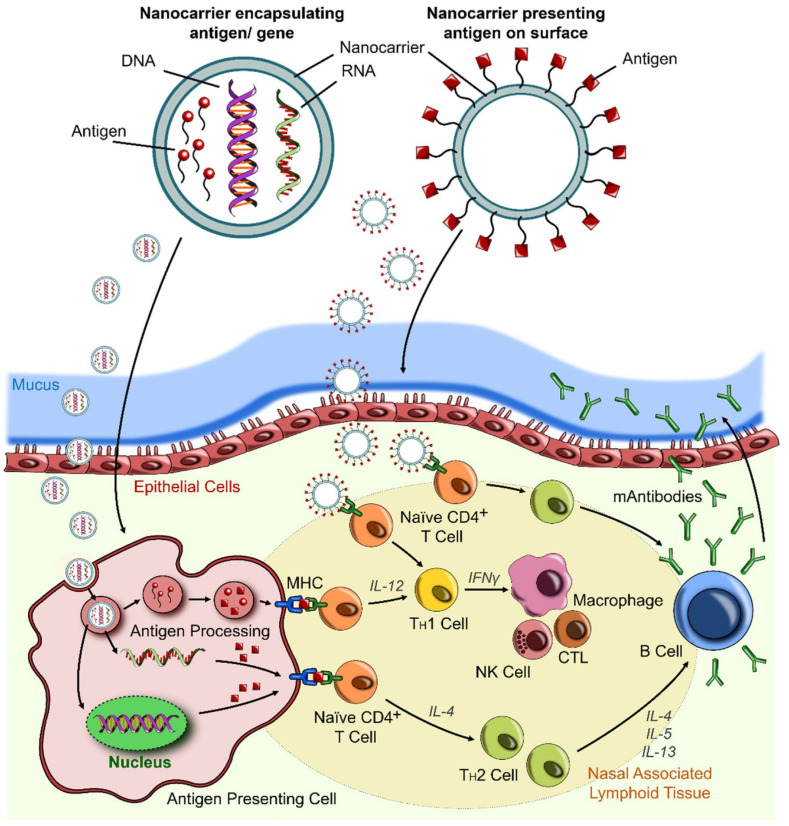
Schematic of providing nano-vaccines based on loading strategy; Nanoparticle-based vaccines that encapsulate an antigen for delivery and trigger an immune. Nanoparticle-based vaccines that have an antigen embedded in their surface. Reproduced with permission from [26]. Copyright Elsevier, 2020.

**Figure 7:**
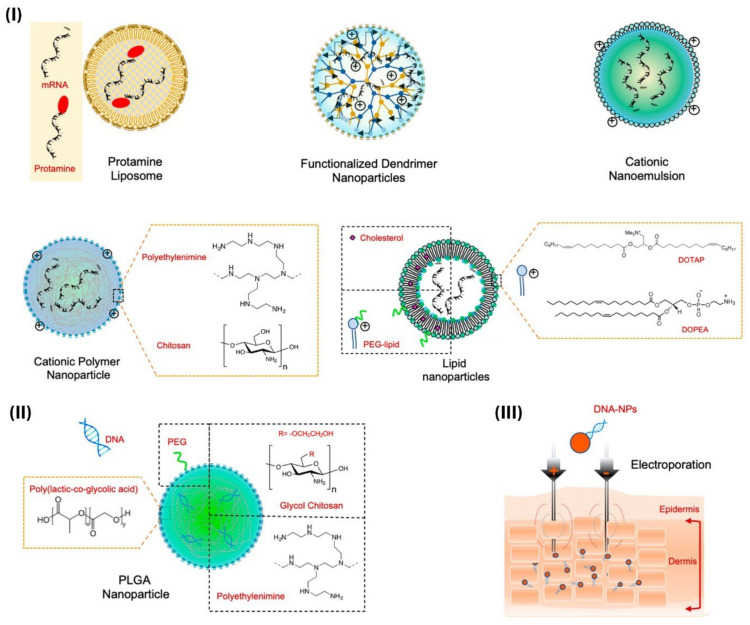
Nano-delivery methods for mRNA and DNA vaccines; (**I**) common carriers for delivery mRNAs, (**II**) PEG based-nanocarriers for DNA delivery, (**III**) electroporation technology for DNA delivery. Reproduced with permission from [112]. Copyright American Chemical Society, 2020.

Strategy 1—Antigen delivery: To better understand the immune system about the properties of viruses, nanoparticles can be used to load a wide range of antigens. Nanoparticle-based delivery systems are a very good alternative in vaccinology compared to conventional delivery methods, which in addition to protecting the antigen structure, also improves antigen delivery and presentation of antigen to the antigen-presenting cells (APCs) [126,127,128]. Antigen loading in two ways: loading on the surface or inside the nanocarrier (Figure 6). Various factors such as physicochemical properties of nanoparticles and antigen, target location, release, and biological stability determine the type of antigen loading [31]. Non-covalent interactions and surface charge are the basis of the physical adsorption of antigens on the surface of nanoparticles. Nanoparticles such as carbon nanotubes, inorganic nanoparticles, polymer nanoparticles are excellent candidates for surface loading of antigens with amphoteric nature [113,129,130,131]. The release of antigens loaded on the nanoparticles takes place in the presence of stimuli such as pH, temperature, and ionic strength. Antigen encapsulation is mainly done to protect against biodegradation. Poly(lactide-co-glycolide) (PLGA) nanoparticles are ideal for encapsulating antigens due to their controlled, long-term release [132]. Antigens with long-term cellular and humoral immune response applications are mainly considered as carrier loads, COVID-19 mRNA-based vaccines that use lipid-based nanoparticles as carriers are very promising [133]. Self-assembled lipid nanoparticles are virus-sized synthesized by an ionized cationic lipid [134]. Extracellular RNases can degrade naked mRNAs, so it is necessary to encapsulate naked mRNAs [135,136]. mRNAs contain ligands that, in addition to detecting target cells, are able to penetrate the carrier lipid membrane. Exogenous mRNAs are converted to functional proteins due to the presence of cytosols. Thus, nanocarriers are designed to enter the cytoplasm to deliver mRNAs efficiently [137]. Sustained release of mRNAs leads to protein translation that provides high antibody titers and both T cells and B cells immune responses [138]. To increase the half-life of these carriers, PEG-lipid and cholesterol are used to reduce clearance and increase stability, respectively. The most common carriers for delivery mRNAs are protamine nanoliposomes, functionalized dendrimer, cationic nanoemulsion, cationic polymer, and lipid nanoparticles (Figure 7I) [139,140,141].

In DNA-based vaccines, bare DNA must be encapsulated to prevent biodegradation by nucleases and effective delivery to specialized immune cells. Nanocarriers such as natural and synthetic polymers, cationic lipid, and inorganic carriers are among the nanocarriers used to carry bare DNA [112]. Composite PLGA polymeric nanocarriers are the most common polymeric carriers for the development of DNA-based vaccines that enhance antigen-specific antibody responses [142,143]. In general, polymer-based carriers have a high ability to encapsulate and prevent biodegradation, controlled release, and targeted delivery. Other polymer-based nanocarriers, such as chitosan and PEI/complex nanoparticles, are used to design DNA-based vaccines [144,145]. Using PEG functionalization on nanocarrier surfaces reduces clearance, reduces toxicity, improved stability, and prevents nonspecific protein interaction (Figure 7II) [146]. Various technologies, including electroporation and gene gun, are used to improve mRNA/ DNA transport in cells and nuclear membranes [147,148]. At present for DNA-based vaccine, for entering DNA into the cell uses electroporation technology to create pores in the cell membrane (Figure 7III). The use of this technology in pigs based on surface electroporation DNA coated-PLGA nanoparticles for efficient cell delivery has been demonstrated to elicit B cell and T cell responses [148].

Strategy 2—Vaccine adjuvant nanoparticles (VANs): VANs are considered to improve efficiency and increase immune response. The use of VANs can also provide other important applications such as reducing the required antigen dose and allowing more production in the current pandemic [149]. Accordingly, among the nano-based vaccines in the preclinical stage, five protein subunit vaccines have been introduced that are a combination of adjuvant and antigen [112,116]. Among these, NVX-CoV2373 is a successful vaccine that will soon enter clinical trials. NVX-CoV2373 is a nanoparticle-based on recombinant SARS-CoV-2 glycoprotein with Matrix M as an adjuvant that has improved the immune response and could be commercially successful as a COVID-19 vaccine [112,116]. The main mechanism of VANs is to inform immune cells using immune-serving cues to the protective immune response against a specific antigen, which is referred to as danger signals [150]. Danger signals are derived from viruses, known as damage-associated molecular patterns (DAMPs) and pathogen-associated molecular patterns (PAMPs) [151]. DAMPs and PAMPs are recognized by specific receptors (pattern recognition receptors (PRRs)) that are expressed by immune cells to release inflammatory cytokines to strongly prepare B and T cells priming [152,153,154]. In particular, adjuvants signal the body’s immune system to develop a tolerance for incoming antigen. VANs also have other applications such as systemic distribution, reduction of clearance rate, lack to targeting immune cells, and preventing the accumulation of antigens in a unique environment [129]. Among VANs vaccines, polymeric nanoparticle-based VANs that encapsulate small molecules target lymphoid organs in a controlled release for specific delivery. VANs, which are based on liposomal nanoparticles, mainly contain lower doses of antigens and cyclic dinucleotide (adjuvant; agonist of INF gene stimulator) and show an uncompromised and safe immune response [155]. In general, if the target VANs are lymph nodes, there will be a significant reduction in dose, while if the target is dendritic cells may require a higher dose than adjuvanticity [112]. In vivo studies showed that co-encapsulating both adjuvants and antigen in calcium phosphate nanoparticles and PLGA, in addition to increasing antigen uptake, also led to high antibody titers and activation of APC [156,157]. Other studies have reported the activation of dendritic cells, followed by T cells, with the possibility of localization of antigens and adjuvants by co-encapsulation strategy in the endosome and phagosome compartments [158,159]. VANs including gold nanoparticles are also used to codeliver immunoregulatory drugs or self-antigens as adjuvants for block autoimmune response [160,161].

#### 2.3.2. Design and Administration Considerations of Nanomedicine-Based Vaccines

**Figure 8:**
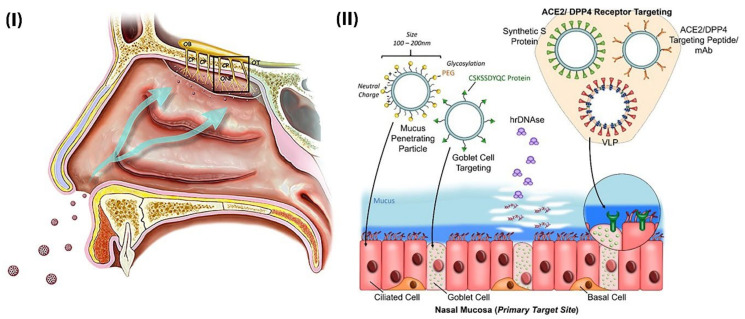
(**I**) Schematic of the route of entry of SARS-CoV-2 and nanoparticles into the nasal cavities and entrapment in the mucosal layer [167] (Reproduced with permission from [167]. Copyright Frontiers, 2020), and (**II**) Including mucosal penetrating nanoparticles for direct targeting of ACE2/DPP4 receptors are nanocarriers based on targeting peptides, targeting ligands (synthetic S proteins) and targeting antibodies. Reproduced with permission from [26]. Copyright Elsevier, 2020.

It has been proven that the main route of entry of SARS-CoV-2 into the host body is through the nasal cavities. Therefore, mucosal epithelial cells such as ciliated cells and mucus-producing goblet cells are the main sites of SARS-CoV-2 infection [26,162]. The Infection is cleared in these areas by nasal-associated lymphoid tissue (NALT) (which includes B cells, T cells, dendritic cells, and macrophages cells). Clearance begins by antigen-specific antibodies and activated killer cells if cells in these areas are stimulated, so an attractive and promising target for the design of vaccines against SARS-CoV-2 could be based on NALT [163,164]. Ideally, nanoparticle-based vaccines against SARS-CoV-2 should follow the same path as viruses to achieve NALT. These vaccines must be designed to have the same kinetics as the virus in the host body. The design can be based on physicochemical properties such as size, shape, and surface load (Figure 8) [165,166].

Apart from the important aspects of nano-vaccine design in targeting the site of infection, the administered route of nano-vaccines is also very important. In order to overcome the aversion to the vaccine and to patient compliance, approaches have been proposed to replace the routes of invasive administration (intravenous and intramuscular) that are non-invasive and painless, such as inhalation, oral administration, and microneedle injection [163]. Recently, many efforts have been made in the development and design of non-invasive nano-vaccines. Mucosal nano-vaccines show an improved immune response by targeting the mucosal immune system and protecting their payloads against degradation and mixing mucosal adjuvant with vaccine [168]. Clinical trials have also reported successful oral (Virus-like particles) and intranasal (viral vectors) delivery of nano-vaccines [169]. Natural nanoparticles have also been suggested for oral immunization due to their high oral bioavailability, stability, and compatibility with the gastrointestinal tract [170,171]. For intranasal delivery of H1N1Me2 and HBsAg, gold nanoparticles and alginate-coated chitosan nanoparticles were synthesized [172,173]. One of the major problems with many conventional vaccines which may reduce patient acceptance is the need for booster doses. Therefore, needle-free nanopatches have been proposed as a suitable method to accelerate vaccine delivery and reduce the burden on healthcare systems [17]. In single-dose vaccines that require slow release, the use of nano-based implants, intranasal-based nano-vaccines, and thin-film-based nano-vaccines may be appropriate options [174,175].

The distribution of conventional solution-based vaccines worldwide is logistically difficult due to the need for constant refrigeration in many less developed countries. Therefore, the development of long-term stable nanotechnology-based vaccines can reduce the need for a cold-chain process. For example, carriers made from cowpea mosaic virus have the ability to protect cargo for more than an hour around 60 °C, and indefinitely at ambient temperature [176]. Recent advances in nanotechnology in the development of vaccines can maintain their temperature-independent stability and establish a good immune response.

## 3. Diagnosis

To end the pandemics, especially the current one, the WHO and other organizations working in the field have concluded that early detection to prevent further spread can lead to the end of pandemics. So, prompt diagnosis and identification of infected patients as soon as possible in pandemics are important. However, due to the unavailability of diagnostic kits, slow output, and sampling, as well as the need for a specialist, conventional diagnostic methods are very slow and difficult. Various methods have been introduced to detect COVID-19, but developing a rapid and sensitive test is still very challenging. 

The first step in diagnosing a person with COVID-19 is based on the person’s travel history, whether or not they have been in contact with patients with COVID-19. If the person’s association with the patient is confirmed, along with mild symptoms, a second stage Chest X-Ray and CT is recommended to detect bilateral shadows and glass opacity in the lungs [177]. The third step in virus detection is a real-time RT-PCR test based on enzymatic detection of SARS-CoV-2 RNA in the throat and nasal swabs, sputum, and bronchoalveolar lavage fluid. The real-time RT-PCR test identifies the structural proteins of the virus, including Spike (S) glycoprotein, Nucleocapsid (N), and Envelope (E), as well as RNA-dependent RNA polymerase (RdRp), in the first open reading frame [178]. Positive readings indicate confirmation of infection and the patient can be a carrier. However, negative results cannot reject the possible diseases [179,180]. Therefore, clinicians recommend CT scans for people who have negative results in RT-PCR screening [181]. However, this may not be appropriate as the virus can affect anywhere in the body due to the properties of COVID-19, so the absence of infection in the lungs cannot be a definite sign of non-infection [19]. Serological tests are another diagnostic method used to detect the presence of Immunoglobulin G (IgG), Immunoglobulin M (IgM), and Immunoglobulin A (IgA) against SARS-CoV-2 S and N proteins [182]. This method is used for COVID-19 rapid detection formats; however, it has some limitations. Another analysis on blood serum methods, such as complete blood count (CBC), lactate dehydrogenase (LDH), C-Reactive Protein (CRP), and AST/AL can be a prognosis of the disease for the patients [177]. Thermal scanners are another technique to detect COVID-19. Thermal scanners only detect high skin temperatures, which can be quite different from body temperature, so they cannot detect a person’s fever. Hence this method is to identify an ambiguous idea [8].

Given the importance of early detection and poor performance of current diagnostic methods, nanotechnology-based targeting pathogens and molecules is a promising strategy in the early and rapid detection of COVID-19. Rapid nanotechnology-based detection can reduce the economic burden and prevent exacerbations.

### Nano-Based COVID-19 Detection

**Figure 9:**
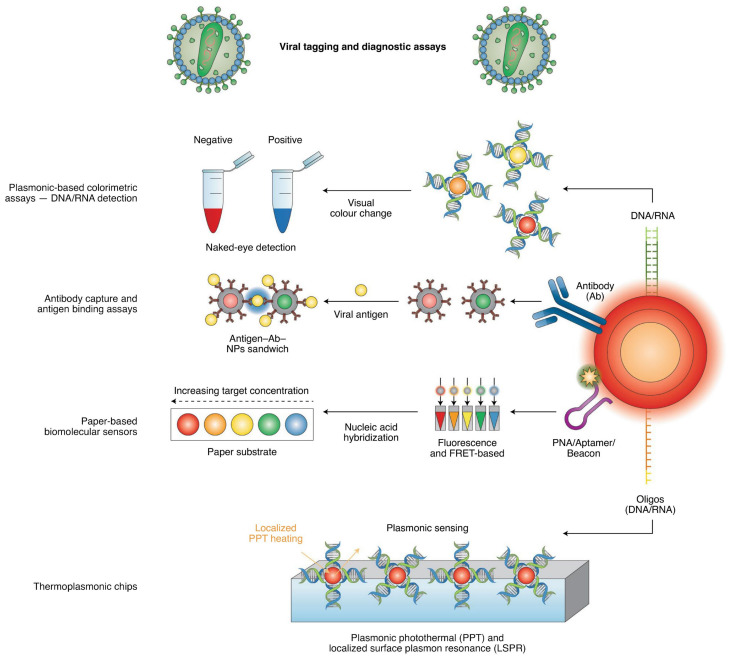
Detection based on colorimetric, antigen binding as well as light and photothermal platforms strategy by nanomaterials engineered with nucleic acids or antibodies is nano-based diagnostic main lines. Reproduced with permission from [191]. Copyright Royal Society of Chemistry, 2020.

Nanoparticles are very effective in detecting high-resistance microorganisms and can be used as an alternative method for the rapid diagnosis of infectious diseases. Various nanotechnology-based devices have been developed that are used to detect the COVID-19 (Table 4). These devices are known as Points of Care devices which are used for diagnosis at a point where the patient is receiving treatments in the diagnostic centers, hospitals, and clinics [183,184]. These devices are based on nanotechnology, which is mainly based on color change, capture and binding, and plasmonic sensing (Figure 9) [6,183,185]. These devices are very popular in the current pandemic due to their high biocompatibility, thermal, electrical, and fluorescence conductivity, and other features [186].

**Table 4:** Detection platforms for pathogenic infections.

Detection Platform	Pathogens	Assay Time	Limitations	Sample Matrix	Ref.
Rapid detection systems	SARS-CoV-2, Influenza virus	13 min	~48% false negatives	Nasal or throat Swab	[187]
RT-PCR	SARS-CoV-2, SARS-CoV, MERS-CoV	48 h	Laboratory equipment, trained personnel, limited to the detection of nucleic acids	Serum, nasal or throat swabs	[188]
Magnetic nanosensors	SARS-CoV-2, Influenza virus	30 min	Laboratory equipment, trained personnel	PBS, serum, water, nasal swab, milk	[125]
CRISPER-Cas method	SARS-CoV-2, Influenza virus	1 h	Laboratory equipment, trained personnel	PBS, serum	[189]
Nanopore Target sequencing	SARS-CoV-2	10 h	Laboratory equipment, trained personnel	PBS, serum	[184,190]

Nanoparticles can be used in any of the points of care devices to detect coronavirus according to different characteristics and applications. Among the most important diagnostic nanoparticles are magnetic nanoparticles, gold nanoparticles, quantum dots, and carbon nanotubes.

Magnetic nanoparticles: Magnetic nanoparticles are widely used in the detection of pathogens due to their properties such as small size, excellent magnetic properties, and high biological performance. The most common magnetic nanoparticles used to target microorganisms such as viruses are superparamagnetic nanoparticles [192]. Magnetic nanoparticles can play a role in the isolation, for example, a one-step nucleic acid extraction for special specifically bind viral RNA developed by Zhao et al. [193] by applying a magnetic field, the nucleic acid was easily collected and released from the nanoparticles by the addition of elution buffer. In one study, superparamagnetic nanoparticles were prepared with a probe to target the cDNA of SARS-CoV [194]. The supra-magnetic nanoparticles in the presence of a magnetic field extract the target cDNA from the sample, which enhances PCR amplification, and it can easily detect the virus [194]. In another study, magnetic nanoparticles activated for RNA extraction were reported for potential COVID-19 detection by Somvanshi et al. [195]. Magnetic nanoparticles were functionalized with carboxyl-modified polyvinyl alcohol and silica which showed the ability to extract RNA in different virus samples. These platforms can reduce the operation steps of detection, which offers great potential for detecting COVID-19 molecular-level.

Gold nanoparticles (AuNPs): AuNPs are the most common metal used to detect coronavirus. AuNPs have been developed for two reasons: (i) Color change and surface plasmon resonance shift, and (ii) ease of electrostatic surface decoration with various antibodies and antigens [196,197]. Color change of Au NPs bound to antibodies is a recognized method without the need for expensive equipment to detect viral proteins. Kim et al., used a disulfide bond-based colorimetric assay, and thiolated ssDNA probes to target and detect the MERS-CoV genome [198]. Similarly, through ssDNA-specific hybridization and target DNA sequence, a SARS-CoV colorimetric hybridization method has been developed [199]. The enzyme-linked immunosorbent assay (ELISA) together with the nanoparticles form a special complex with the collision and a third set with the nanoparticles, which results in immobilization and accumulation of the nanoparticles, which cause shifts the color from red to blue [200]. Such low-cost methods are found in countries with limited medical resources and developing countries. Recently, biosensor devices have been used for on-site detection, which works with a specific antibody against the SARS-CoV-2 spike protein [201]. Furthermore, plasmonic photothermal (PPT) and dual-functional plasmonic biosensors have been combined as a cost-effective detection method which is a fast alternative to RT-PCR [202].

Quantum dot: The most widely used quantum dots to detect the COVID-19, which is known as a new fluorescent probe for molecular imaging. The unique properties of Quantum Dots such as optical properties and easy control of their emission wavelength make them a great candidate as a fluorescent label [203,204]. Therefore, they are currently used as imaging probes in the form of chemical sensors and biosensors for sensing [205]. Ashiba et al. [206], For example, introduced a biosensor to detect viruses with a highly sensitive that uses the Quantum Dot fluorescent dye to assay. In this sensor, by optimizing the intensity of autofluorescence of the substrate on the chip, it detects the accumulation of 100 virus particles. In another study, a combination of Quantum Dots-conjugated RNA aptamer-based chip was used to detect highly sensitive and rapid SARS-CoV N protein [207].

Carbon-based nanomaterials: Recently, carbon-based materials have been widely used in COVID-19 diagnostic platforms [208]. Carbon nanotubes are carbon-based nanoparticles that typically have high biocompatibility and stability and have a variety of applications, including biosensitivity and bioimaging [209,210]. Recent studies of the applications of carbon nanotubes for the detection of respiratory viruses, including SARS-CoV and SARS-CoV-2, have been reported. Yeh et al. [211], For example, using a novel CNT size-tunable enrichment microdevice (CNT-STEM) to detect the virus that channel sidewall was lined with nitrogen-doped multiwalled carbon nanotubes. The distance between the carbon nanotubes is optimized to match the size of different viruses. This platform enhances detection sensitivity and virus isolation rates. Hence CNT-STEM well-detect influenza virus. Furthermore, this platform can be used to detect SARS-CoV-2 proteins or RNA due to the ease and reliability of this technique [211]. Carbon nanotubes have also been used in optical sensors because they have a high affinity for binding to SARS-CoV-2 S proteins. In fact, a single-wall carbon nanotube (SWCNT) can improve the fluorescence signal in the presence of target viruses [212].

## 4. Treatment

**Figure 10:**
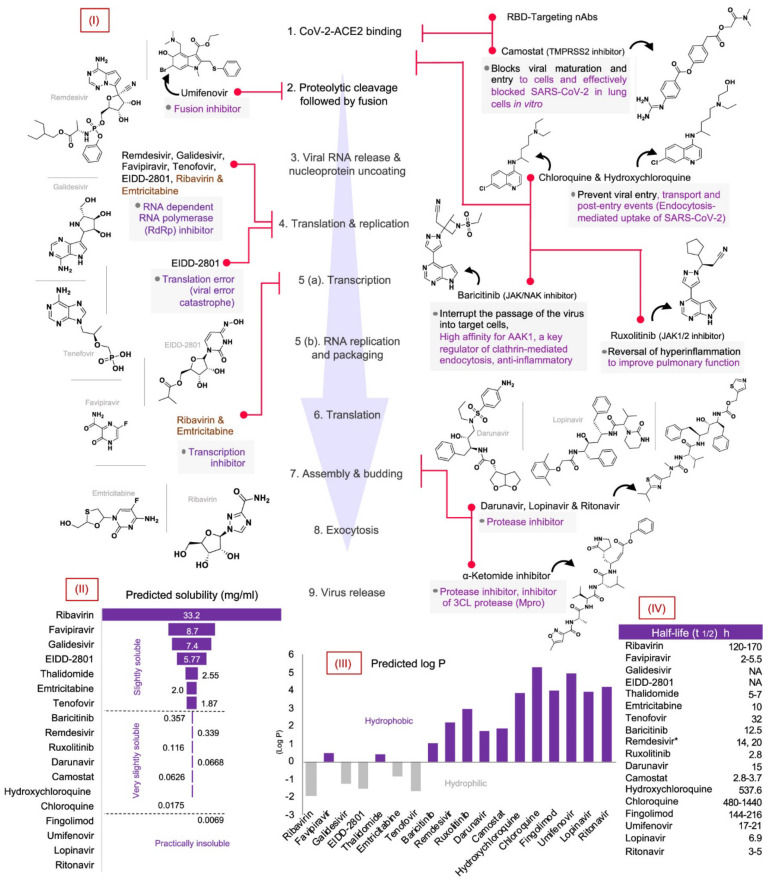
Antiviral molecules for COVID-19 therapeutics [112] (Reproduced with permission from [112]. Copyright Royal Society of Chemistry, 2020.); (**I**) mechanism, chemical structure and site of antiviral action, (**II**,**III**) solubility and log P, and (**IV**) half-life (t1/2) of antiviral molecules.

COVID-19 treatment strategies are being developed and their clinical success has not been reported. Therefore, current treatments for the COVID-19 virus in individuals are based on symptomatic therapies. Recently, using modeling of the structure of virus proteins and genetic information, several therapeutic approaches based on drug repurposing for faster treatment of infected have been proposed. Identifying targets is the most important step in achieving successful treatment in COVID-19. In the treatment of SARS-CoV-2, the most important therapeutic targets are, viral protease (3CLpro and PLpro), RNA polymerase (RdRp), and prevent viral and protein interactions with host receptor ACE2 (Figure 10) [213,214,215,216,217].

Neutralizing antibodies are used to target the SARS-CoV-2 S protein, which is a promising treatment group to prevent the progression of infection [218]. In fact, antibody neutralization has the ability to block protein S to prevent binding to Dipeptidyl-peptidase 4 (DPP4) and ACE2 receptors MERS-CoV, SARS-CoV, and SARS-CoV-2. Targeting the siRNA genome is another attractive treatment for SARS-CoV-2, which uses antisense oligonucleotides or RNA interference [219]. siRNAs are designed to accurately target the SARS protein M mRNA with an interference efficiency of more than 70% [220]. Despite unknown RNA sequence domains for the SARS-CoV-2, RNAi (including RNA aptamers, and small interfering RNAs) can be used to treat SARS infection [219]. Another approach currently used in clinical trials is the soluble human ACE2 called APN01 [221]. Disruption of ACE/ACE2 in patients with COVID-19 increases angiotensin II (Ang II) levels, leading to severe lung injury and ARDS. APN01 is able to lower Ang II levels by completely covering the virus surface, thus balancing ACE/ACE2 [221,222].

In general, therapies that target the SARS life cycle can be promising, but the development of these methods is very slow and may take years to complete. Hence, maybe they not reach the commercialization stage for unexpected pandemics like COVID-19. Therefore, the use of nanotechnology capabilities for targeted drug delivery and adjunctive therapies can be very promising.

### 4.1. Nano-Based Approach for COVID-19 Treatment

In addition to the applications of nanoparticles in the development of vaccines, they can also be used in new therapeutic applications to combat COVID-19. Nanomedicine approaches are mainly used to reduce toxicity and side effects as well as to remove limitations related to therapeutic agents. At present, various treatment techniques are being developed using nanotechnology, some of which are discussed below.

#### 4.1.1. Targeting ACE2

As discussed elsewhere, due to the presence of ACE2 receptors throughout the body, the COVID-19 virus can infect any of the organs. Therefore, targeting ACE2 to provide therapeutic services to all infected cells can be very promising in preventing the progression of the virus [26]. Targeting can be accomplished through a variety of methods, including the conjugation of targeting agents such as peptides and antibodies on the surface of nanocarriers or using the virus-like particle that contains SARS-CoV-based proteins [223]. Virus-like nanoparticles can be easily transported to mucosal ciliary cells and can also be used for efficient protein delivery to target ACE2. Although it is mainly referred to as the delivery power of virus-like particles, these antiviruses, like smart viruses, are able to escape the immune system. This feature enables them, in addition to transmitting antivirus to the site of infection (lung), to target other sites affected by SARS infection [26]. The ACE2 targeting strategy is greatly facilitated by synthetic S proteins embedded as ligands on the surface of nanoparticles [223,224]. Therefore, after the injection of modified particles by synthetic ligands, they easily bind to ACE2 receptors, so the natural S protein of the SARS virus will not be able to bind to the target cell [225,226]. However, this strategy can be promising in the early stages of detection, before viruses enter cells. Engineering the surface properties of nanoparticles can also facilitate binding to ACE2 receptors. It has been reported that cationic nanoparticles bind easily to the ACE2 receptor, while anionic nanoparticles do not exhibit this behavior [227]. This can be due to the negative charge on the cell membrane. However, cationic nanoparticles do not have high penetration into the mucosal layer and may not be able to block receptors on ciliated cells [26]. Another concern with ACE2 blockade is a disorder of the renin-angiotensin system (RAS) that results from a decrease in the enzymatic cleavage of angiotensin-II, which ultimately increases its blood levels, which subsequently can promote ARDS [228,229]. However, additional targeting and the use of multilayer therapies such as ACE2-targeted delivery of drugs followed by administration of a soluble form of ACE2 (APN01) can overcome these problems. Another problem is reduced drug delivery to infected cells because their receptors are blocked [228,230].

#### 4.1.2. Targeting the Immune System

**Figure 11:**
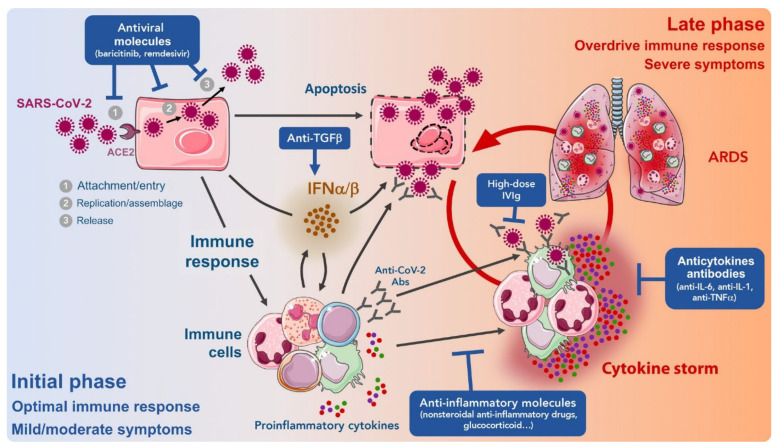
A model of COVID-19-induced infection and possible effective treatments based on enhanced immune response. After SARS-CoV-2 enters the lungs, epithelial cells induce a weak interferon (IFN) α/β production in the initial phase of infection. Hence a limited antiviral immune response is generated which leads to apoptosis of infected cells. This causes the production of pro-inflammatory molecules and the call of immune cells. Using antiviral drugs as a supplement to immunomodulator therapies can reduce the viral load. Reproduced with permission from [231]. Copyright Cell Press, 2020.

As previously discussed, the rapid progression of the disease where a large number of patients experience severe pneumonia is due to a weakened immune system that varies from patient to patient depending on age or other pre-existing conditions. The fact that many SARS-CoV-2-infected patients are asymptomatic suggests that an effective immune response counteracts the virus. When the immune response is ineffective, the disease worsens and may lead to acute respiratory distress syndrome (ARDS). Immune support strategies to further clear and control inflammation caused by an infection in these patients can be helpful and reduce their mortality (Figure 11). 

Anti-inflammatory agents are used to stimulate the immune system, and targeted delivery of anti-inflammatory by nanocarriers can be very promising [26]. Nanocarriers have a high potential for delivering anti-inflammatory drugs to immune cells, including inflammatory macrophages, and T cells to block the production of TNFα, IL-1, IL-6, and other cytokines [232]. Extensive studies have been performed on the maintenance of stability and resistance of anti-inflammatory agents to degradation by nanocarriers. For example, for lung delivery, dexamethasone acetate was loaded into lipid nanoparticles [233]. The delivery of anti-inflammatory agents to macrophages has always faced many challenges [232]. A promising target for specific delivery is the presence of mannose receptors, known as CD206, at the macrophage level [234]. Biodegradable nanoparticles that facilitate localization in the presence of mannose receptors are designed to deliver siRNA against TNFα expression in macrophages [235].

#### 4.1.3. Photodynamic Inactivation of SARS-CoV-2

Photodynamic therapy is a non-invasive method used for therapeutic applications [236]. Photodynamic therapies are primarily used to treat oncological disorders and in some cases against the virus [237]. Photodynamic therapy by stimulating light-sensitive agents such as gold nanoparticles known as photosensitizers, which can produce reactive oxygen species (ROS) in the presence of oxygen at a given wavelength and ultimately lead to cell death [237]. ROS production is used to damage the structural proteins of viruses [238]. Various research efforts have been made on the use of photodynamic therapy and nanotechnology against various viruses, including human immunodeficiency virus, human papillomavirus, and herpes simplex virus [238,239,240,241]. However, photodynamic therapy faces various challenges due to its limited hydrophobicity and poor penetration depth. The effects of photosensitizers on photobiological and photochemical properties in aqueous solutions have led Lim et al. [242] to offer a promising method for photodynamically inactivating viruses by responsive nanoparticles. The modification of nanoparticles with phthalocyanine photosensitizers onto their surfaces, in addition to increasing hydrophilicity, also shows strong antiviral activity. Nano-graphene and fullerene are also excellent candidates for inactivation of viruses by photodynamic therapy, that applications are against the Semliki Forest virus (SFV), vesicular stomatitis virus (VSV), and influenza A virus (IAV) [238]. Photobiomodulation and photodynamic therapy are also used to reduce the cytokine storm caused by COVID-19, which can directly act on the chest area. Photodynamic therapy is also acted on the bone marrow to further increase the synthesis of stem cells and immune-modulate [243]. Therefore, photodynamic therapy-based approaches combined with nanotechnology can be very promising for inactivating viruses such as SARS-CoV-2.

#### 4.1.4. Nano-Based Delivery of Therapeutic Agents

**Figure 12:**
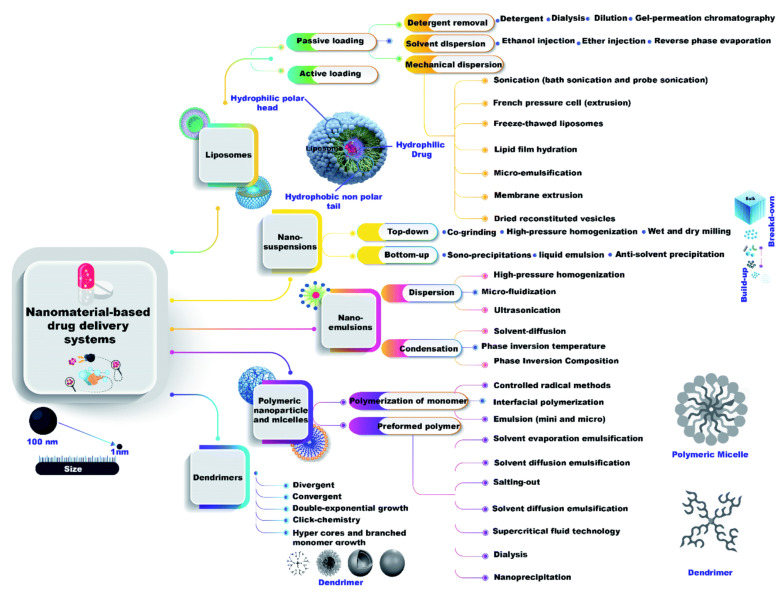
The most common nanomaterials in drug delivery applications and their preparation methods. Reproduced with permission from [34]. Copyright American Chemical Society,2020.

Side effects of antiviral drugs have led to the development of nano-based drug delivery systems. In free injection of drugs, they can damage non-infected cells, while higher doses can cause some organs to become ineffective. The unique properties of nanocarriers such as good solubility, biocompatibility, long circulation time, as well as surface performance, make them an excellent candidate for the delivery of therapeutic agents [28,244,245]. Different types of nanocarriers have been introduced for drug delivery applications (Figure 12). Nanoparticles face various biological barriers after injection, the first and most important of which is the removal of nanoparticles from circulation. There are natural processes for the clearance of foreign substances, including nanoparticles, by organs such as the kidneys, spleen, liver, as well as immune cells that reside in various organs [246]. On the other hand, coating the surface of nanoparticles with serum proteins after injection causes the formation of a protein corona. The protein corona helps the mononuclear phagocyte system to easily identify particles. This causes endocytosis of particles by these systems and removes them from the circulation. Removal of nanoparticles from the circulation by these mechanisms reduces their bioavailability, which also reduces their therapeutic efficacy. The ability to manipulate the physicochemical properties of nanoparticles allows these barriers to be overcome [247]. Furthermore, surface modifications of nanoparticles by ligands with specific targets, it is possible to significantly prevent the attachment of nanoparticles to normal cells and subsequently the toxicity caused by the particles [28]. 

Given the widespread use of nanocarriers in the delivery of antiviral agents in the treatment of viral infections, it is predicted that they may also be successful in the treatment of COVID-19. Therefore, studies in the field of a combination of nanotechnology and pharmacy for the treatment of COVID-19 have accelerated. Some of the most important therapeutic drugs of COVID-19 are hydroxychloroquine (HCQ) and chloroquine (CQ). Although the mechanism of their effectiveness and the actual extent of their effectiveness are still unknown, they have yielded promising results. However, its high side effects in long-term use, especially in high doses, are associated with serious risks, for example, for hydroxychloroquine, an increased risk of heart disease has been reported [248]. Recently, a computational study based on the simulation of molecular dynamics of hydroxychloroquine loading on the surface of gold and silver nanocarriers has been investigated [249]. In this study, it was found that high-efficiency hydroxychloroquine molecules can be loaded. Hence, by designing the safe and optimal nanocarriers for the delivery of hydroxychloroquine, the treatment of COVID-19 can be helped with minimal side effects [249]. Another study showed that multidrug-loaded nanocarriers could reduce the controlled inflammation caused by COVID-19 infection [250]. To control excessive cytokines, α-tocopherol and adenosine were loaded into squalene nanoparticles as an antioxidant and an immune regulator, respectively. These nanoplatforms are transported to SARS-CoV-2 infection sites to relieve inflammation and reduce cytokines. In addition, by protecting their cargo, they prevent their unwanted side effects [250].

Among the most important nanoparticles in virus treatment applications are metal nanoparticles and organic nanoparticles:

Metal nanoparticles: These nanoparticles have a high loading capacity that in addition to drug delivery activities also has antiviral activities. These nanoplatforms are typically used to deliver the antiviral drugs ribavirin (RBV) and oseltamivir (OTV) [251]. Delivery of OTV by selenium nanoparticles is able to inhibit enterovirus 71 (EV71) by reducing ROS generation in an EV71-infected U251 cell line [252]. RBV-loaded selenium nanoparticles have also been used to treat MERS-CoV, SARS-CoV, and influenza virus strains [253,254]. For example, these nanoplatforms (Se@RBV) with a diameter of 65–100 nm have been used to reduce the titer of the influenza H1N1 virus through resisting the caspase-3 pathway [255]. It was found that levels of DNA damage, lung injury, perivascular edema, and peribronchiolar were significantly lower in in vivo (H1N1 influenza infected BALB/c mice) samples using Se@RBV intranasal injection than in uncontrolled groups [255]. Se@RBV is able to reduce oxidative stress in cells, so it can be effective in COVID-19 patients with reported oxidative stress [255]. Furthermore, gold nanoparticles are able to remain in different types of cells and tissues for a long time due to their compatibility and permeability [256]. Therefore, they have been used to deliver RBV to destroy the measles virus in African green monkey kidney cells (Vero cells) [257]. Nanocomposites consisting of silver and graphene oxide nanoparticles, in addition to their antiviral activity against enveloped and non-enveloped viruses (FCoV and IBDV), also have the ability to control release [258]. For example, the controlled release of ions contributes to the production of ROS, which in addition to stimulating drug release, is a potential antiviral agent for enveloped and non-enveloped viruses. Virus inhibition assays have shown that silver and graphene oxide composites are able to reduce FCoV and IBDV infection by 25% and 23%, respectively. While these nanoparticles alone have not shown significant ability to fight these viruses [259]. It was found that the delivery of antiviral drugs by gold nanoparticles had a much stronger antiviral effect compared to free injection.

Organic nanomaterials: Organic nanomaterials, including polymers and lipid nanoparticles, which are composed of renewable nanomaterials, have been very promising in drug delivery applications [260,261,262,263]. For example, sustained release of two antivirals, diphyllin, and bafilomycin, as vacuolar ATPase inhibitors by dual-block PEG-PLGA copolymers, reduced the viral titer in the lungs of a mouse model, increasing its viability by 33% [264]. Lipid-based nanoparticles can release antiviral drugs into cell membranes. Lipid-based nanoparticles can also be used for intravenous or intranasal injections to enhance the effect of antiviral agents. In some cases, injecting them can greatly affect treatment efficiency [265]. For example, intranasal injection of the liposome-acyclovir formulations increased the adhesion of the mucosal adhesion by 60.7% compared to intravenous injection and increased drug absorption in the nasal cavity [266], which is the main site of respiratory infections such as COVID-19. However, fat-based nanoparticles have limitations, including their limited load capacity, especially for hydrophilic drugs [267].

#### 4.1.5. Gene Editing

Various viruses including respiratory viruses can cause persistent disease by integrating their genome into the host cell genome [268]. Therefore, direct targeting of the viruses’ RNA and DNA genes can be very effective in eradicating viruses and stopping their persistent infections [17,269]. The CRISPR/Cas technology is able to target RNA and DNA genomes directly with high accuracy and power in both the pre-integration and provirus stages, which is a promise for controlling the current pandemic, SARS-CoV, and pandemic influenza [270,271]. SARS-CoV-2 is able to alter membrane proteins by mutations in its genetic sequence, thus reducing the effect of antiviral drugs and antibodies. Therefore, instead of focusing on membrane proteins, virus replication can be prevented by targeting RNA genomes of SARS-CoV-2 and destroying them using CRISPR/Cas systems [272]. Recently, a study was reported to be able to cleave the SARS-CoV-2 RNA genome based on the CRISPR/Cas system. The system is based on a gRNA-containing sequence that uses an adeno-associated virus to package Cas13d protein and gRNA to improve effectiveness. Some adeno-associated virus serotypes display a highly-specific tropism for lung tissue, so they can enter gRNAs into only lung cells, so it is a successful strategy to fight the SARS-CoV-2 without affecting the transcriptome of the lungs. However, genetic modification of the SARS-CoV-2 virus may also lead to the emergence of new strains that can resist the CRISPR/Cas system, thus increasing the virulence of the virus. It is therefore critical to target multiple genomic sites [273,274].

Concerns about CRISPR/Cas systems are their instability at the serum nucleases, unwanted stimulation of the immune system, and its clearance by the kidneys [275,276]. Therefore, designing CRISPR/Cas systems based on nanoparticles can be effective for treatment programs. For example, lipid-based nanoparticles have been used to co-load the streptococcus pyogenes Cas9 (SpCas9) mRNA and modified single-guide RNA (sgRN) in mouse models. In addition to stability and biodegradability, this nanosystem is able to edit the transthyretin (Ttr) gene in the liver of mice for one year about 97% [277]. In another study, lipids-based materials were used to deliver CRISPR/Cas to knock down the proprotein convertase subtilisin/kexin type 9 (Pcsk9) gene. After intravenous injection, this leads to reduce the expression of HBV DNA [278].

## 5. What Is the Role of In Silico Models?

**Figure 13:**
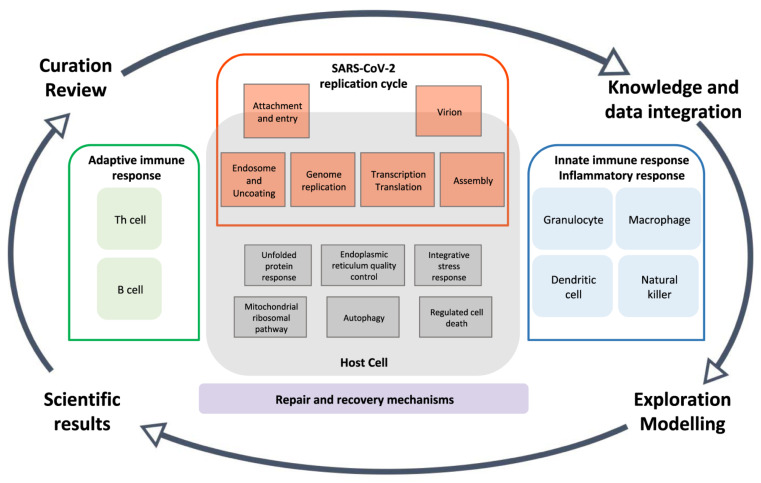
COVID-19 Disease Map; The focus of this map is on SARS-CoV-2 interactions with the host, SARS-CoV-2 replication cycle, and reaction of the immune system and repair mechanisms. Content interacts continuously with databases to support disease modeling, and support visual and computational exploration. Reproduced with permission from [285]. Copyright Springer Nature, 2020.

**Figure 14:**
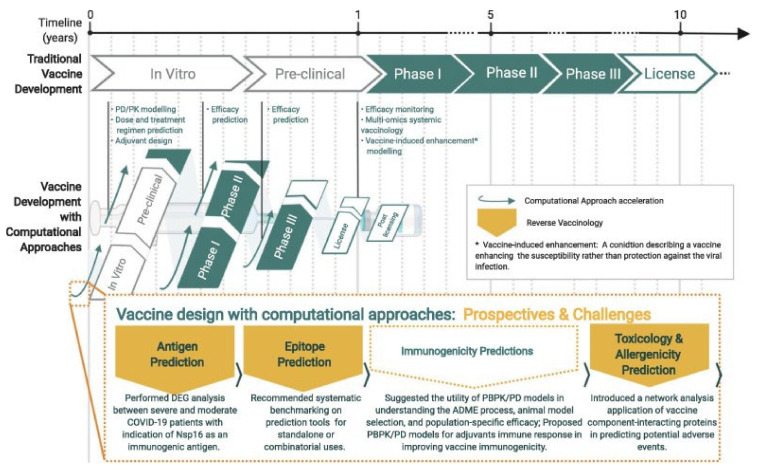
Computational tools can accelerate the development of vaccines at various stages. The center of the diagram shows what role computational models can play at each stage of vaccine development. Reproduced with permission from [291]. Copyright Elsevier, 2021.

Recently, computational models have been used as an adjunct to in vivo and clinical models in various fields of medicine [279,280,281,282]. In the current pandemic, computational models also play a significant role [283,284]. Therefore, projects have been defined in this field that has led to the preparation of a map of COVID-19 disease (Figure 13) [285]. This map was developed in order to gain in-depth knowledge of the mechanism and interactions between the host and the SARS-CoV-2, which is considered a document for the development of high-quality models. In recent years, advances in computational genomics and molecular biology have led to a better understanding of protein structures and their function in host cells and viruses, which has effectively facilitated the design of antiviral drugs. Computer simulations have made it possible to design nucleotide inhibitors through the application of structural information and mutational analyses [286]. Computational fluid dynamics simulations also make it possible to identify contaminants and spread them, which has led to the optimal design of clean rooms in the current pandemic [287,288,289,290]. Computational models can also be used to help develop prophylactic vaccines. While vaccine development typically takes about 10 years, computational models can not only reduce costs but also reduce this time and speed up the development process (Figure 14) [291]. Recently launched vaccines have mainly used computational models at various stages such as antigen prediction [291]. Computational methods have also been used to predict and recommend T cell epitope as one of the targets of the SARS-CoV-2, vaccine [292]. In addition to guiding the vaccine, computational models are also used to understand the immune response of recovering patients.

In the computational approach, which is currently used as an alternative to many other models, molecular dynamics, computational chemistry, and molecular docking are among the most important tools used in pre-clinical and clinical studies [282,293]. Given the promising performance of nanomedicine and its urgent need against the current pandemic, computational analysis can be very useful in the logical designing of nanocarriers for drug delivery and gene delivery applications. A combination of large databases and computational techniques makes it possible to select the best drug candidate. A better understanding of nano-bio interactions has led to computational models having a significant impact on the creation and development of clinical translation-based systems [279]. Recently, ACE2-based peptides have been designed using classical molecular dynamics simulations [294]. It was found that the multivalent binding of peptides on the surface of nanoparticles, lead to improvement of the binding affinity of ACE2 for peptides increased. Furthermore, regarding the COVID-19 to provide useful guidance for nanoformulations against SARS-CoV-2, the cellular internalization of gold nanoparticles has been evaluated by simulating the coarse-grained molecular dynamics to investigate the targeting mechanisms of intracellular delivery.

The design of computational models should be done with the support of the literature, expert opinions, the Association of Pharmacists and Physicians, and, if possible, based on clinical and preclinical findings. After selecting the best therapeutic agent candidates from computational analysis, in vivo trials should be performed prior to clinical trials, although computational models can be a complement to in vivo and clinical models.

## 6. Concluding Remark

Despite the rapid advancement of our understanding of the COVID-19 virus and its mechanism of action, we have learned that we need to learn more.. In fact, one of the important lessons of the current pandemic is to show that expeditious research is possible. This virus challenges us in different ways, so it forces us to be more creative in our approach and, of course, rely on scientific standards.

In the current study, we discussed the coronavirus and the current pandemic and reviewed ways to prevent, diagnose, and treat based on nanotechnology, and tried to clarify the good, the bad, and the ugly. Accordingly, the coronavirus plays the bad role in this issue, and the infections caused by it play the ugly role. While engineered nanoparticles play the good role. It should be noted that “nano” is only a scale of size on which biological interactions take place, so “nano” is neither good nor bad. Importantly, an understanding of biological interactions at this scale to manipulate nano-bio interactions may provide a new way to prevent and treat infectious diseases. Hence, “nano” is just a toolbox. The story of the “coming of age” of nanoparticles for decades has been of interest to researchers and is different from what was expected. When the first nanoparticles were approved in 1995, many believed that nanomedicine would revolutionize diagnosis and treatment. However, many studies on the diagnosis and treatment of cancer refer to it as “evolving technology.” In fact, complex pathology and proving its effectiveness is still difficult, an obstacle that Pfizer and Moderna have not overcome because they have only tried to get emergency permits for their vaccine.

Recently, due to the need to quickly end the COVID-19 pandemic in the world, the results of the effectiveness of new therapeutic agents are announced every day. Meanwhile, advances in the field of nanotechnology have put it in the spotlight, and this shows that nanotechnology can be a cornerstone for addressing various health challenges in the future. However, nanomedicine acts as a double-edged sword. On the one hand, it can increase the bioavailability of drugs, increase the effectiveness of drugs, inhibit virus binding, inhibit inflammation, and inactivate the virus in and out of the body. On the other hand, there are concerns about its toxicity. There have been several reports of mutagenesis, production of free radicals, penetration into the brain, etc. for nanoparticles, especially metal nanoparticles. Therefore, special research should be done regarding ease of administration, dose size, biodegradability, and toxicity caused by nanoparticles. Another point is cell death by the stimulation of nanoparticles that react against pathogens. In the current pandemic, there is so much unknown about SARS-CoV-2 that using the wrong particle with minimal side effects can lead to adverse effects on public opinion that cause panic throughout the nanosystem.

Despite the development of different types of nanoparticles for therapeutic and diagnostic agent delivery applications in the treatment of various diseases, including cancer and COVID-19, their clinical translation is still an important problem. This is because many candidates fail in clinical trials. The clinical failure of nanoparticles in the first stage is related to preclinical models that are not sufficiently developed. Some important biological differences between animal and human models not only confound clinical translation but also lead to the cessation of many clinical trials. For example, the immunotoxicity of nanoparticles, especially metal nanoparticles, has led to the cessation of many clinical trials, whereas this toxicity has not been found in animal models. Therefore, the standardization of preclinical studies is very important for the evaluation performance of nanomedicine. Preclinical models for clinical translation should be such as to provide more accurate predictions of nano-bio interactions. Meanwhile, computational models and artificial intelligence as a tool to optimize the physicochemical parameters of nanoparticles based on biological parameters, are able to improve preclinical models that increase the chances of clinical translation. On the other hand, computational models can significantly reduce the need for animal experiments. The second stage of clinical failure of nanoparticles can be attributed to the lack of sufficient confidence to invest in this field. This has led to many preclinical studies not being developed for clinical trials due to a lack of funding [247]. Many researchers believe that the epidemic of COVID-19 becomes a chronic and seasonal disease. Therefore, it is necessary to invest continuously in the development of treatment methods and vaccines. Candidates for SARS-CoV and MERS-CoV vaccines did not enter the market due to lack of financial incentive and a low number of infected, so the global pandemic caused by a new virus was ignored. Therefore, at the present time, it is necessary to continue research and product development to deal with any new version of the coronavirus that may appear in the future. We anticipate that nanoparticles will make great strides in development and treatment in the future, but perhaps the biggest nanoparticle challenge, clinical translation, remains.

## Figures and Tables

**Table 1:** Sterilization and disinfection of inanimate surfaces based on nanotechnology.

Developer	Structure	Characteristics	Ref.
SHEPROS SDN BHD	A nano silver-based multipurpose disinfectant using a nano-colloidal technique	Environmentally friendly, non-irritating, and non-foaming properties to fight viruses, germs, and fungi	[56]
Nano Tech Surface	A nano-sterilizing based on silver ions and titanium dioxide	Environmentally friendly, non-irritating, High disinfection potential	[57]
Weinnovate Biosolutions	A nano-sterilizing based on silver nanoparticle solution	Low toxic effects, High disinfection potential, non-irritating,	[58]
Tamil Nadu University	A natural nanomaterial-based disinfectant containing hydrogen peroxide and alcohol molecules	High environmental friendliness, non-corrosive, non-toxic, cost-effective	[8]
Defence Institute of Advanced Technologies (DIAT) in Pune	A disinfectant called Ananya based on a water spray using nanomaterial	Able to adhere to fabric, plastics, and metal surfaces, and disinfection effects about 6 months.	[59]
Design.123	A disinfect based on nanopolymer	High disinfection potential, Fast performance in inactivating viruses	[60]
Hong Kong University of Science and Technology	A temperature-responsive nanopolymer-based antimicrobial	Slowly releasing disinfectants, high lifespan	[61]
EnvisionSQ	An antiviral nanocoating	High lifespan, high ability to kill viruses	[62]
NanoTouch Materials	A nano-coating based on mineral nanomaterials	High disinfection potential, non-corrosive	[8]

**Table 2:** Nano-based disinfectant personal protective equipment (PPE).

Developer	Structure	Characteristics	Ref.
Promethean Particles Ltd.	A fabric based on copper nanoparticles embedded in a polymer matrix	Enhancing antiviral and antimicrobial properties	[85]
ZEN Graphene Solution Ltd.	silver-nanoparticle-modified graphene oxide nanocomposite membranes	Virus capture and killing, virucidal	[86]
Sonovia Ltd.	A fabric based on zinc oxide nanoparticles	Antiviral properties, and can be washed for reuse	[86,87]
Master Dynamic Limited	A coating based on nanodiamonds	High anti-virus performance	[86]
X.TiO2 Inc. (XTI)	TiO_2_Ag-based facemasks	Able to kill 99.99% of viruses under zero light conditions	[86]
Verdex Technologies Inc.	Nanocomposite membranes	Virus removal, breathable	[86]
Respilon	Nanofiber membranes	High virus removal	[88]
Yamashin-filter Corp.	Polymer-based nanofiber membranes	High virus trapping, virus removal	[89,90]
LIGC Application Ltd.	Graphene-based technology	Reusable, self-sterilizing, antiviral activity	[86]
Balagna et al.	Silver nanoclusters/silica composite sputtered coating	Virucidal, increased lifetime of masks and filter media	[91]

**Table 3:** The role of nanotechnology in vaccine developments.

Strain of Coronavirus	Vaccine	Nano Component	Mechanism	Ref.
Influenza virus	Pulmonary surfactant biomimetic nanoparticles	Biomimetic liposomes	Potentiate heterosubtypic influenza immunity	[116]
MERS-CoV	Purified coronavirus spike protein nanoparticles	Spike nanoparticles	Induce coronavirus neutralizing antibodies in mice	[117]
SARS-CoV	Gold nanoparticle-adjuvanted S protein	Gold nanoparticles	Induced antigen-specific IgG response	[118]
	SARS subunit vaccine	Peptide nanoparticles	Neutralizing antibody and strong humoral response	[119]
SARS-CoV-2	Novel lipid nanoparticle (LNP)- encapsulated mRNA based vaccine	Lipid nanoparticles	Recombined mRNA of the S protein	[120]
	LNP-encapsulated mRNA encoding RBD	Lipid nanoparticles	RBD mRNA reacted strongly with a SARS-CoV-2 RBD specific antibody	[121]
	LNP-nCoV-saRNA	Lipid nanoparticles	Robust neutralization of a pseudovirus, proportional to quantity of specific IgG	[122]
	Self-replicating RNA based therapeutic vaccine (LUNAR-COV19 STARR™)	RNA nanoparticles delivery systems	Enhanced adaptive cellular (CD8+ cells) and balanced (Th1/Th2) immune response	[123]
	virus-like nanoparticles (VLNP)	Protein nanoparticle scaffold	Promotes B cell immune response	[124]
	T-COVID	Adenovirus	Decreased cellular inflammation and lower concentrations of IL-6	[125]

## Data Availability

The study did not report any data.

## Abbreviations

WHO	World Health Organization
CT	Computed tomography
DIAT	Defence Institute of Advanced Technologies
ACNSMM	Amrita Centre for Nanosciences and Molecular Medicine
APCs	Antigen-presenting cells
DAMPs	Damage-associated molecular patterns
PRRs	Pattern recognition receptors
RdRp	RNA-dependent RNA polymerase
IgM	Immunoglobulin M
CBC	Complete blood count
CRP	C-Reactive protein
ELISA	Enzyme-linked immunosorbent assay
RT-PCR	Real-time polymerase chain reaction
STEM	Size-tunable enrichment microdevice
DPP4	Dipeptidyl-peptidase 4
ARDS	Acute respiratory distress syndrome
SFV	Semliki Forest virus
IAV	Influenza A virus
CQ	Chloroquine
OTV	Oseltamivir
SpCas9	Streptococcus pyogenes Cas9
Ttr	Transthyretin
ACE2	Angiotensin-converting enzyme 2
PPE	Personal protection equipment
HKUST	Hong Kong University of Science and Technology
RBD	Receptor-binding domain
PLGA	Poly(lactide-co-glycolide)
PAMPs	Pathogen-associated molecular patterns
NALT	Nasal-associated lymphoid tissue
IgG	Immunoglobulin G
IgA	Immunoglobulin A
LDH	lactate dehydrogenase
AuNPs	Gold nanoparticles
PPT	Plasmonic photothermal
CNT	Carbon nanotubes
SWCNT	Single-wall carbon nanotube
RAS	Renin-angiotensin system
ROS	Reactive oxygen species
VSV	Vesicular stomatitis virus
HCQ	Hydroxychloroquine
RBV	Antiviral drugs ribavirin
EV71	Enterovirus 71
sgRN	Single-guide RNA
Pcsk9	Proprotein convertase subtilisin/kexin type 9
